# Asymptotically Unbiased Estimation of Exposure Odds Ratios in Complete Records Logistic Regression

**DOI:** 10.1093/aje/kwv114

**Published:** 2015-09-30

**Authors:** Jonathan W. Bartlett, Ofer Harel, James R. Carpenter

**Keywords:** complete case analysis, logistic regression, missing data, odds ratio

## Abstract

Missing data are a commonly occurring threat to the validity and efficiency of epidemiologic studies. Perhaps the most common approach to handling missing data is to simply drop those records with 1 or more missing values, in so-called “complete records” or “complete case” analysis. In this paper, we bring together earlier-derived yet perhaps now somewhat neglected results which show that a logistic regression complete records analysis can provide asymptotically unbiased estimates of the association of an exposure of interest with an outcome, adjusted for a number of confounders, under a surprisingly wide range of missing-data assumptions. We give detailed guidance describing how the observed data can be used to judge the plausibility of these assumptions. The results mean that in large epidemiologic studies which are affected by missing data and analyzed by logistic regression, exposure associations may be estimated without bias in a number of settings where researchers might otherwise assume that bias would occur.

Missing data are a pervasive issue in epidemiologic studies, reducing statistical power to detect associations of interest and potentially biasing estimates. In a recent systematic review of studies using questionnaires in leading epidemiologic journals, over 90% suffered from missing data to some extent (1). There now exist a plethora of statistical techniques for analyzing partially observed data sets, including multiple imputation (2–4) and inverse probability weighting (5). However, the most commonly used approach (1) is complete records analysis (CRA), often referred to as complete case analysis.

Whether estimates are biased by missing data depends on the relationships between the chance of data being missing and the variables involved in the analysis. Many readers will be familiar with the acronyms MCAR, MAR, and MNAR, first developed by Rubin (6) for classifying the mechanisms causing data to be missing (7). It is well known that if data are missing completely at random (MCAR), meaning that missingness is independent of the variables involved in the analysis, CRA is asymptotically (i.e., in large samples) unbiased, and that generally it is biased if data are not MCAR. However, it is perhaps less widely appreciated that CRA can be asymptotically unbiased for some or all of the parameters in certain situations where data are missing at random (MAR) or even missing not at random (MNAR) (8–10).

Whether a CRA produces asymptotically unbiased estimates depends both on the type of missingness mechanism present and on the form of the analysis to be undertaken. In this article, we focus on the common situation where the analysis consists of fitting a logistic regression model for a disease outcome, with an exposure of interest and a number of confounders as covariates. Logistic regression is a popular approach for modeling binary outcome data, for a number of reasons. First, in case-control studies, in which participants are sampled with a probability that depends on the disease outcome, logistic regression permits valid estimation (although the intercept parameter has a different interpretation from that in a cohort study). Second, the logistic function is the canonical link function for a binary outcome, so logistic regression enjoys certain favorable properties.

In the present article we review and extend earlier results (10–12), which we have found to be rather neglected recently, showing that CRA estimates of exposure odds ratios from logistic regression are asymptotically unbiased under a surprisingly wide range of missingness mechanisms. We then provide guidance as to how the observed data can be used to investigate the missingness mechanism, thereby indicating whether the CRA exposure odds ratio estimate is likely to be asymptotically unbiased. The results have important implications for the analysis of epidemiologic studies suffering from missing data: In some settings where researchers might believe a CRA is biased due to data not being MCAR, a CRA logistic regression analysis may in fact produce asymptotically unbiased exposure odds ratio estimates.

We first describe the conditions under which CRA logistic regression gives asymptotically unbiased estimates of the exposure odds ratio, which slightly extends the previous results (10–12). Next, we describe a set of simulations based on data from a cohort study of professional flight crew in the United Kingdom, illustrating these results. Third, we give detailed guidance for how to use the observed data to draw conclusions about the missingness mechanism(s) and thereby to help decide whether CRA logistic regression will produce an asymptotically unbiased exposure odds ratio estimate. We conclude with a discussion.

## VALIDITY OF EXPOSURE ODDS RATIO ESTIMATES FROM CRA LOGISTIC REGRESSION

### The outcome model

To describe when a CRA logistic regression analysis will produce asymptotically unbiased estimates of the exposure odds ratio, we assume that in the population of interest the binary outcome *Y* (typically representing the occurrence of an event of interest) follows a logistic regression given exposure *X* and confounders *C*:

(1) $$\text{logit}(P(Y=1|X,C))=\beta_{0}+\beta_{X}X+\beta_{C}C.$$

We assume that data are available from a set of study participants. If the data come from a cohort study, in the absence of missing data we obtain asymptotically unbiased estimates of the population values of β_0_, β*_X_*, and β*_C_*. If the data are from a (unmatched) case-control study, as is well known, estimates of the adjusted covariate associations β*_X_* and β*_C_* are asymptotically unbiased, but the intercept β_0_ depends on the sampling fractions of cases and controls (13).

### Missingness

We now suppose that for some participants, data for one or more of the variables involved in the logistic regression are missing. We let *R* denote a binary indicator of whether a participant has *Y*, *X*, and *C* observed and thus has a complete record (*R* = 1) or an incomplete record (*R* = 0). CRA then consists of fitting the logistic regression model to the complete records, that is, using data from those participants for whom *R* = 1. Whether CRA produces asymptotically unbiased estimates depends on the dependence of the binary indicator *R* on the variables *Y*, *X*, and *C*. Note that we do not specify which variables suffer from missingness—the results that follow are not dependent on where the missingness occurs, and therefore, as noted by Westreich (10), they apply regardless of whether or not the data are MAR or MNAR.

If *R* is independent of *Y*, *X*, and *C*, the data are MCAR. In this case, the complete records form a random subsample of the target sample, so any parameters which would have been estimated without bias in the absence of missing data will continue to be estimated without bias.

Unfortunately, data are rarely MCAR in epidemiologic studies, and thus an important question is what types of missingness lead to bias in the CRA estimate of the exposure odds ratio. In a previous paper published in the *Journal*, Vach and Blettner (11) considered the case of a binary exposure and a single partially observed categorical confounder and described missingness conditions under which the CRA estimate of the exposure odds ratio from logistic regression is asymptotically unbiased. In fact, their results apply more generally, and they apply irrespective of whether it is the outcome, the exposure, or the confounder which has missing values. The following results also have links to those of Hernán et al. (12), who considered the issue of selection bias from a causal diagram perspective, although for the most part they considered a setting more general than the logistic regression setting considered here; the symmetry of the logistic link means that some results that hold for logistic regression do not apply in general. Westreich (10) also used causal diagrams to discuss selection bias and bias due to missing data, emphasizing that the odds ratio can be asymptotically unbiased when other association measures are biased, although his presentation considered a setting with no confounders.

We now describe the situations in which the CRA estimates of β*_X_*, and sometimes the estimates of β_0_ and/or β*_C_*, are asymptotically unbiased. The results are summarized in Table 1. Derivations are given in Web Appendix 1 (available at http://aje.oxfordjournals.org/).

**Table 1.** Bias of Estimates Derived From Complete Records Analysis Logistic Regression Under Different Missingness Assumptions

Quantity on Which Missingness Is Dependent	Parameter		
	β_0_	β*_X_*	β*_C_*
Neither *Y* nor *X* nor *C*	Asymptotically unbiased	Asymptotically unbiased	Asymptotically unbiased
Outcome (*Y*)	Biased	Asymptotically unbiased	Asymptotically unbiased
Covariates (*X*, *C*, or both)	Asymptotically unbiased	Asymptotically unbiased	Asymptotically unbiased
Outcome (*Y*) and confounders (*C*)	Biased	Asymptotically unbiased	Biased
Outcome (*Y*), exposure (*X*), and possibly confounders (*C*)	Biased	Biased^a^	Biased

^a^ Biased in general. However, if *P* (*R* = 1|*X*, *Y*, *C*) = *s* (*X*, *C*)*t* (*Y*, *C*) for some functions *s* (*X*, *C*) and *t* (*Y*, *C*), with *R* being the complete record indicator, then the exposure association is again estimated without bias (asymptotically).

### Outcome-dependent missingness

If missingness depends only on the outcome, that is, *P*(*R* =1|*X*, *Y*, *C*) = *P*(*R* = 1|*Y*), estimates of β*_X_* (and β*_C_*) are asymptotically unbiased, while estimates of the intercept are biased. This result is, of course, the primary reason that the odds ratio is used as an association measure in case-control studies (14), in which selection into the study corresponds to having a complete record, with the probability of selection depending on case/control status.

### Covariate-dependent missingness

CRA is also asymptotically unbiased under covariate-dependent missingness, that is, *P*(*R* = 1|*X*, *Y*, *C*) = *P*(*R* = 1|*X*, *C*). In fact, this result applies not only to logistic regression but more generally to any regression method in which the model specifies the distribution of outcome *Y* given covariates *X* and *C* (e.g., linear regression) (15). Intuitively the result can be understood by the fact that when one fits a regression model for an outcome *Y* given covariates *X* and *C*, one is only estimating some aspect (e.g., the mean) of the distribution of *Y* separately in different strata defined by the covariates. While covariate-dependent missingness means the complete records are not a random sample, it ensures that (in cohort/cross-sectional studies) within strata defined by the covariates, the distribution of *Y* is representative of the stratum-specific population distribution of *Y*.

### Missingness dependent on the outcome and a confounder

Suppose now that missingness depends on *Y* and *C* but given these is independent of *X*, that is, *P*(*R* = 1|*X*, *Y*, *C*) =*P*(*R* = 1|*Y*, *C*). Then CRA is again asymptotically unbiased for β*_X_* (but generally is biased for β_0_ and β*_C_*). Intuitively this result holds, because when we adjust for *C*, the exposure association can be thought of as being estimated separately in strata defined by *C*. When missingness depends on *Y* and *C*, the stratum-specific odds ratio for exposure is not altered (in expectation), such that we again obtain asymptotically unbiased estimates of β*_X_*.

### Missingness dependent on the exposure and the outcome

In general, if missingness depends jointly on *X* and *Y* (and possibly *C*), CRA is biased for β*_X_*. Intuitively this can be understood by the fact that such mechanisms affect (within the complete records) the distribution of exposures differently in persons with *Y* = 1 from those with *Y* = 0.

There is, however, a class of mechanisms in which missingness depends jointly on *X* and *Y* but for which CRA is still asymptotically unbiased for β*_X_*. Specifically, this is the case if *P*(*R* = 1|*X*, *Y*, *C*) = *s*(*X*, *C*)*t*(*Y*, *C*) for some functions *s*(*X*, *C*) and *t*(*Y*, *C*) (10). This result can be viewed as following from combining the preceding results on covariate-dependent missingness and missingness dependent on the outcome and a confounder in turn. Moreover, *s*(*X*, *C*) might depend only on *X*, so that *s*(*X*, *C*) = *s*(*X*), and *t*(*Y*, *C*) might depend only on *Y*, so that *t*(*Y*, *C*) = *t*(*Y*).

Missingness mechanisms satisfying this condition might arise in practice when missingness occurs in 2 variables (or sets of variables), such that to have a complete record a participant must have both variables observed. For example, suppose that participants with the outcome of interest are more difficult to follow up to have their outcome observed, such that missingness in *Y* is dependent on *Y*. Next, suppose that the exposure is less likely to have been observed for those participants with high exposure levels. In this scenario, CRA would be asymptotically unbiased for β*_X_*, with *P*(*R* = 1|*X*, *Y*, *C*) = *s*(*X*)*t*(*Y*) for suitable functions *s*(*X*) and *t*(*Y*), which respectively determine how the probabilities that *X* and *Y* are observed depend on *X* and *Y*. Notice that here data would be MNAR, yet the exposure odds ratio is still estimated without bias (asymptotically).

### Interaction

Thus far, we have assumed a model with main “effects” of exposure *X* and confounders *C*. In some settings, one or more components of *C* may act as modifiers for the association of *X* with *Y*, such that interest lies in a logistic regression model which includes interactions between *X* and one or more components of *C*. All of the preceding results still apply in this case, where instead of a single exposure odds ratio β*_X_*, the logistic regression model estimates stratum-specific exposure odds ratios, with strata defined by combinations of one or more of the components of *C*.

### Model misspecification

The results described above assume that the logistic regression outcome model is correctly specified. In practice, models will generally be misspecified to some extent. For example, the confounders *C* may be included in the model in the incorrect functional form, or the exposure association may in truth vary with a component of *C*, while in the outcome model we assume no interaction. The question then arises as to whether missingness biases our estimates relative to the values which would have been unbiasedly estimated (asymptotically) in the absence of missing data.

Unfortunately the preceding results do not apply in general when the outcome model is misspecified. For example, in the case where interaction is present but is not included in the outcome model, the estimated exposure odds ratio in the full data is an average association, averaged over the distribution of the confounders. Performing a CRA means averaging across the confounder distribution of the complete records, which will generally differ from the confounder distribution in the full data, resulting in an estimate which is biased as an estimate of the population “parameter” that would be estimated in the absence of missing data. However, provided that the outcome model is only mildly misspecified, we would expect any such biases to be small and therefore for the results to hold approximately. This is illustrated empirically below. In cases where the outcome model is severely misspecified, the “parameters” being estimated in the absence of missing data are of questionable use, so additional biases caused by missingness are arguably of secondary concern.

### Results for other types of regression models

Thus far, we have considered bias for exposure associations in logistic regression. As we noted above, covariate-dependent missingness does not cause bias in CRA more generally (15), including in linear regression for continuous outcomes and Cox proportional hazards models for time-to-event outcomes. Furthermore, it is known that Cox proportional hazards models produce estimates similar to those of logistic regression when the follow-up period is the same for all participants and the event rate is low (16). This approximate equivalence implies that when follow-up is the same across participants and the event rate is low, the results regarding when CRA logistic regression produces asymptotically unbiased exposure association estimates also apply to Cox regression, with the outcome *Y* corresponding to the event indicator. Thus, provided that follow-up is similar across participants and the event rate is low, a CRA Cox regression would be expected to give approximately asymptotically unbiased exposure association estimates when *P*(*R* = 1|*X*, *Y*, *C*) = *s*(*X*, *C*)*t*(*Y*, *C*).

## ASSESSING THE PLAUSIBILITY OF MISSINGNESS ASSUMPTIONS IN PRACTICE

In practice, we must use a combination of exploratory data analysis and contextual knowledge to judge the plausibility of different missingness assumptions. Suppose that either a subset of the confounders *C*_1_ (where *C* = (*C*_1_, *C*_2_)), the exposure, or the outcome is partially observed. In each case a logistic regression model can be fitted in which the dependent variable is a binary indicator of whether the partially observed variable(s) is (are) recorded or missing, and the independent variables are the remaining fully observed variables in the outcome model. The results of this logistic regression model for missingness can inform the plausibility of the different missingness assumptions. Table 2 summarizes what might be reasonably concluded from this analysis regarding the missingness mechanism, and hence whether CRA logistic regression would be expected to produce an asymptotically unbiased estimate of the exposure odds ratio. Web Appendix 2 gives a detailed explanation of these results and provides guidance for the common situation in which more than 1 variable is partially observed.

**Table 2.** Guidance for Investigation and Implications of Missingness Mechanisms in Complete Records Analysis Logistic Regression

Quantity With Which Missingness Is Found to Be Associated	Plausible Missingness Mechanism(s)	Bias in CRA Estimate of β*_X_*
*Missingness in a Confounder* C_1_		
*C*_2_	*C*_2_	Asymptotically unbiased
*X*, and possibly *C*_2_	*X* and *C*_2_	Asymptotically unbiased
*Y*, and possibly *C*_2_	*Y* and *C*_2_	Asymptotically unbiased
*X*, *Y*, and possibly *C*_2_	*X* and *Y*	Generally biased
	*C* and *X*	Asymptotically unbiased
	*C* and *Y*	Asymptotically unbiased
*Missingness in the Exposure* X		
*C*	*C*	Asymptotically unbiased
*Y*	*Y*	Asymptotically unbiased
*C* and *Y*	*C* and *Y*	Asymptotically unbiased
	*X* and *Y*	Generally biased
	*X* and *C*	Asymptotically unbiased
*Missingness in the Outcome* Y		
*X*	*X*	Asymptotically unbiased
*C*	*C*	Asymptotically unbiased
*X* and *C*	*X* and *C*	Asymptotically unbiased
	*Y* and *C*	Asymptotically unbiased
	*X* and *Y*	Generally biased

Abbreviation: CRA, complete records analysis.

We note that when investigating the missingness mechanism, one should not focus solely on statistical significance—in small studies, strong mechanisms may not reach statistical significance due to lower power, while in large studies an association between missingness and a variable might be statistically significant yet sufficiently small in magnitude to deem any effect on bias negligible. We also emphasize that the guidance given as to which missingness mechanisms are plausible in light of analysis of missingness cannot be definitive. In particular, for the class of mechanisms where CRA is biased (joint dependence on *X* and *Y*), it is possible to construct situations where associations cancel out in such a way that the missingness may appear to belong to one of the other (non-bias-causing) classes. This points to the importance of contextual knowledge, together with appropriate sensitivity analysis (17), in applications.

## ILLUSTRATIVE ANALYSIS

### Study data

To illustrate the preceding results, we analyzed data from a cohort study of professional flight crew (pilots, flight engineers, and navigators) in the United Kingdom (18, 19). The study aimed to include all United Kingdom residents who held a professional flight crew license at some point between 1989 and 1999, and it made use of the Medical Records System of the United Kingdom Civil Aviation Authority. In total, 16,327 flight crew members were recruited into the study. For the analyses presented here, the start of follow-up was defined as the date of issuance of the first valid license in the medical record database for each crew member. The Medical Records System contains data from routine health surveillance examinations given to each crew member every 6 or 12 months.

Flight crew are subject to a number of occupational exposures which may potentially be related to increased risk of adverse outcomes. For the illustrative analyses presented here, we used the data to estimate the association between each crew member's number of flying hours at baseline, as a proxy for the crew member's exposure to cosmic ionizing radiation, and his or her subsequent risk of death in the following 15 years. Confounders adjusted for were age, sex, smoking status, body mass index (BMI; weight (kg)/height (m)^2^), and type of flight route (no commercial flights, United Kingdom, Europe, or world). Baseline exposure and confounder information was obtained from the health examination corresponding to the crew member's date of the first valid license (issued during the study period). We then linked crew members' records to the United Kingdom's health registers to obtain vital status information up to 2006. The exposure (*X*) incurred during each crew member's number of accrued flying hours at baseline was categorized as 0 (<400 hours), 1 (400–5,499 hours), or 2 (≥5,500 hours), using the original cohort study's tertiles. We adjusted for age using a piecewise linear function with knots at 30, 40, 50, and 60 years, while BMI was categorized with cutpoints at 20, 25, and 30.

Some individuals (*n* = 3,585) were censored for vital status prior to 15 years, although the majority of these persons (*n* =2,616) had follow-up of at least 10 years. Proper analysis of the data required this to be accounted for using survival analysis techniques. For the purposes of the illustrative analysis presented here, we ignored censoring and took as the outcome whether each individual was observed to die during his or her follow-up period of up to 15 years. Of the 16,327 persons followed, 354 died during follow-up. Information on exposure was missing for 42 individuals, route type was missing for 43, and 643 had missing BMI. Following the strategy outlined in the previous section and expanded on in Web Appendix 2, we ignored the small proportions of missing values in the route-type and number-of-flight-hours variables and focused on investigating missingness in BMI using a logistic regression model for the indicator of its missingness, including the outcome, exposure, and other confounders as covariates. This showed that age, route type, number of flying hours, and sex were related to missingness in BMI but that conditional on the exposure and other confounders, there was no evidence of an association between missingness in BMI and the outcome. An assumption of covariate-dependent missingness was thus plausible, such that the CRA estimate of the exposure association (and indeed other covariate associations) should have been asymptotically unbiased. After dropping the 644 individuals missing data on BMI, route type, or number of flying hours, the data set contained 15,683 records. We refer to this as the “full data,” since, as we describe below, we next made further data artificially missing in order to explore the extent to which the theoretical results were borne out when applied to a real data set.

### “Full data” estimates and CRA estimates under different missingness mechanisms

The first row of Table 3 shows the log odds ratio estimates for the associations of 400–5,499 flying hours versus <400 hours and ≥5,500 flying hours versus <400 hours with mortality, adjusted for the confounders, based on the “full data” from 15,683 aircrew. Next, for 8 different missingness mechanisms, we simulated a complete record indicator *R* for each study participant and fitted the logistic regression model using the resulting complete records. This was repeated 10,000 times for each of the 8 missingness mechanisms. Table 3 shows the mean of the exposure log odds ratio estimates across the 10,000 repetitions, the mean standard error, and the percent bias for each mechanism. For each mechanism, the overall probability of having a complete record was approximately 0.5, except for the last mechanism, for which the probability was 0.25. Except for the MCAR mechanism, each mechanism assumed a reasonably strong dependence for the probability of having a complete record on one or more of the death-indicator outcome (*Y*), the confounder age (*C*), or the categorical flying-hours exposure (*X*).

**Table 3.** Log Odds Ratios for the Adjusted Association Between Number of Flying Hours (Categorized) and Mortality Among United Kingdom Flight Crew Members, 1989–1999^a,b^

Missingness Mechanism	Quantity on Which Missingness Is Dependent	*P*(*R* = 1)^c^	No. of Flying Hours			
			400–5,499 vs. <400		≥5,500 vs. <400	
			Log OR (SE)	% Bias	Log OR (SE)	% Bias
	N/A (full data)	N/A	0.64 (0.22)	N/A	0.70 (0.23)	N/A
1	Nothing (MCAR)	expit(0)	0.65 (0.32)	1.3	0.72 (0.32)	2.4
2	Death indicator (*Y*)	1 if *Y* = 1	0.65 (0.23)	1.4	0.72 (0.23)	2.5
		0.485 if *Y* = 0				
3	Age (*C*)	expit((age − 37.32)/10.79)	0.58 (0.29)	−9.0	0.63 (0.27)	−9.9
4	Flying hours^d^ (*X*)	expit(−(flyhrscat − 1))	0.65 (0.28)	0.9	0.72 (0.30)	2.4
5	Age and flying hours (*C* and *X*)	expit(−(flyhrscat − 1) + (age − 37.32)/10.79)	0.60 (0.27)	−6.4	0.64 (0.26)	−9.1
6	Death indicator and age (*Y* and *C*)	expit((age − 37.32)/10.79) if *Y* = 0	0.77 (0.36)	19.1	0.90 (0.42)	28.0
		expit(−(age − 37.32)/10.79) if *Y* = 1				
7	Death indicator and flying hours (*Y* and *X*)	expit(−(flyhrscat − 1)) if *Y* = 0	1.67 (0.40)	160.6	2.76 (0.36)	292.5
		expit(flyhrscat − 1) if *Y* = 1				
8	Death indicator and flying hours (*Y* and *X*), conditionally independently	expit(−(flyhrscat − 1)) if *Y* = 1	0.66 (0.29)	3.5	0.74 (0.31)	5.9
		expit(−(flyhrscat − 1)) × 0.485 if *Y* = 0				

Abbreviations: MCAR, missing completely at random; N/A, not applicable; OR, odds ratio; SE, standard error.

^a^ Simulations based on imposing artificial missingness on data from the Medical Records System of the United Kingdom Civil Aviation Authority. Data were obtained from a cohort study of professional pilots, flight engineers, and navigators who held a professional flight crew license in the United Kingdom at some point between 1989 and 1999 (18, 19).

^b^ Estimates (SEs) from the full data and averages obtained across 10,000 replications under various missingness mechanisms.

^c^ expit(*t*) = exp(*t*)/(1 + exp(*t*)).

^d^ flyhrscat = 0 if flying hours <400, 1 if 400 ≤ flying hours <5,500, and 2 if flying hours ≥5,500.

For data MCAR (mechanism 1), the average estimates were close to those based on full data, as we would expect. With missingness dependent on outcome (mechanism 2), the average estimates of the exposure log odds ratio were again close to the full data estimates. With missingness dependent on age (mechanism 3), both coefficients were reduced somewhat, with downward biases of around 10%. This is likely due to the fact that in the full data, the (adjusted) odds ratio for increasing flying hours was lower in the older crew members, so the outcome model was to some extent misspecified. Under mechanism 3, the complete records had, on average, an age distribution with a higher mean value, and since in older crew members the flying-hours association was somewhat smaller, we saw a slight reduction in average estimated coefficients. For the mechanism dependent on exposure (mechanism 4), the probability of having a complete record increased with decreasing flight hours, and the CRA exposure odds ratio estimates again had little bias.

In mechanism 5, missingness depended jointly on age and flying hours, with complete records being more likely for older participants and those with low flying hours (or both). The net effect of this was that the participants with complete records had a higher mean age, such that exposure odds ratio estimates were somewhat (6% and 9%) lower than those from the full data. In mechanism 6, for those crew members who survived, increasing age increased the probability of having a complete record, whereas in those who died the opposite was true. This meant that among the complete records, the mean age of persons who died was reduced in comparison with the full data, and consequently the exposure log odds ratio estimates were dominated by the associations among the younger crew, which, as previously noted, were larger. This led to upward biases of 19% and 28% for the two log odds ratios.

A dramatic difference was seen for mechanism 7, which depended jointly on the outcome and flying hours (161% and 293% upward biases). Under this mechanism, the CRA suggested that longer flying hours had a much larger association with death than the full data estimates. This was a consequence of our choosing a mechanism in which, among persons who died (*Y* = 1), the probability of having a complete record was set to be higher if the crew member had a higher number of flying hours, whereas in those who did not die (*Y* = 0), this probability was set to be lower if the number of flying hours was higher. In the final mechanism (mechanism 8), missingness depended on outcome and exposure, but independently, such that estimates were again quite close to the full data estimates (upward biases of 4% and 6%).

Overall, consistent with theory, we have seen that estimates are (on average) reasonably close to those from full data under all missingness mechanisms except when missingness depends jointly on outcome and exposure in a nonindependent way. Our results support our view that—in the large majority of situations—substantive conclusions would only be materially affected under missingness mechanisms of this type.

## DISCUSSION

In this article, we have drawn together a number of earlier results to show that for a correctly specified logistic regression model, CRA estimates of exposure associations can be asymptotically unbiased under a surprising range of selection/missing-data mechanisms. Specifically, exposure odds ratios are estimated without bias (asymptotically) provided that missingness does not depend jointly on exposure and outcome, and even then, special cases exist where bias does not result. As Westreich noted previously (10), these conditions apply irrespective of which variable(s) contains missing values, and thus apply whether or not the data are MCAR, MAR, or MNAR.

In epidemiologic studies, we recommend that researchers use both data analysis and contextual knowledge to make judgments about missing-data mechanisms. We have given detailed guidance as to how the observed data can be used to examine the plausibility of different missingness assumptions. However, as we have emphasized, it is essential that such analyses be combined with contextual knowledge in order to judge the overall plausibility of a missingness assumption. A useful tool in this process is the directed acyclic graph, which can encode beliefs about how variables affect each other and missingness (10, 12, 20). Once an appropriate directed acyclic graph has been constructed, standard rules for manipulating such graphs can be used to deduce whether the missingness mechanism falls within one of the classes in which the CRA logistic-regression exposure odds-ratio estimate is asymptotically unbiased.

We emphasize that we are not claiming that CRA will be asymptotically unbiased generally—in the flight crew analyses, we empirically demonstrated the potential for serious biases (mechanism 7). Moreover, for other types of analyses, such as longitudinal studies in which interest lies in estimating the marginal mean of an outcome measure at a particular point in time, CRA is asymptotically unbiased only under very strong (and typically implausible) missingness assumptions.

Even when CRA is asymptotically unbiased, it is not efficient, since it discards the observed data in the incomplete records. In some settings where missingness affects a number of variables in the analysis model, the complete records may constitute only a small proportion of the original sample, such that CRA estimates are very imprecise. If the assumed missingness mechanism satisfies the MAR assumption, multiple imputation can be used to obtain more efficient estimates (2–4). Furthermore, in this case, results from CRA can be compared with those from multiple imputation—material differences may be symptomatic of misspecification in the imputation model, or a sign that the missingness assumptions made by one or another method are violated. If covariates are believed to be MNAR with missingness dependent on the covariates (and given these, independent of the outcome), methods which give more precise estimates than CRA have recently been proposed (21). For more general MNAR mechanisms, Lin and Lyles (22) recently proposed an approach based on collecting a “reassessment sample,” whereby additional data collection is performed to obtain a subset of those exposure values which were originally missing, thus enabling identification of the MNAR mechanism. In the absence of such additional data, whenever there is a nontrivial proportion of missing data and there is doubt regarding the missingness mechanism, sensitivity analyses should be considered (3, 23, 24).

## Supplementary Material

Web Material
