# Supplementary material for: Asymptotically Unbiased Estimation of Exposure Odds Ratios in Complete Records Logistic Regression
Source: Am J Epidemiol. 2015 Sep 30;182(8):730–6. doi: 10.1093/aje/kwv114 (PMC4597800; doi:10.1093/aje/kwv114)
Supplement: Web Material [file supp_182_8_730__index.html]

Asymptotically Unbiased Estimation of Exposure Odds Ratios in Complete Records Logistic Regression — Web Material 

# Asymptotically Unbiased Estimation of Exposure Odds Ratios in Complete Records Logistic Regression

## Web Material

Web Material

- Web Material - Pdf file
