# Supplementary material for: Asymptotically Unbiased Estimation of Exposure Odds Ratios in Complete Records Logistic Regression
Source: Am J Epidemiol. 2015 Sep 30;182(8):730–6. doi: 10.1093/aje/kwv114 (PMC4597800; doi:10.1093/aje/kwv114)
Supplement: Web Material [file supp_kwv114_kwv114supp.pdf]

Web Appendices for "Asymptotically Unbiased Estimation of Exposure Odds Ratios in Complete Records Logistic Regression" by Bartlett et al.

WEB APPENDIX 1

In this appendix we give derivations for the conditions under which the complete records analysis (CRA) logistic regression exposure association is estimated without bias (asymptotically). We assume that the logistic regression model in equation 1 of the main paper is correctly specified. This means there is no interaction between C and X, and so the odds ratio exp(β_X) can be expressed as

$$\exp(\beta_X) = \frac{P(Y=1|X=x+1,C=c)}{P(Y=0|X=x+1,C=c)} \times \frac{P(Y=1|X=x,C=c)}{P(Y=0|X=x,C=c)} \qquad (2)$$

where x and c denote arbitrary values of X and C. CRA consists of estimating the logistic regression model in those participants for whom R = 1. To derive the odds ratio (OR) for X estimated by CRA, consider first the probability in the top left of equation 2. The value of this probability in the complete records is equal to

$$P(Y=1|X=x+1,C=c,R=1)$$

$$= \frac{f(Y=1,x+1,c,R=1)}{f(x+1,c,R=1)}$$

$$= \frac{P(R=1|Y=1,x+1,c)f(Y=1,x+1,c)}{P(R=1|x+1,c)f(x+1,c)}$$

$$= \frac{P(R=1|Y=1,x+1,c)}{P(R=1|x+1,c)}P(Y=1|x+1,c)$$

Using the same argument we can find corresponding expressions for the values of the other three probabilities, conditional on R = 1. The OR estimated for X in a CRA is thus

$$\exp(\beta_X^{CR}) = \frac{\frac{P(R=1|Y=1,x+1,c)}{P(R=1|x+1,c)}P(Y=1|x+1,c)}{\frac{P(R=1|Y=0,x+1,c)}{P(R=1|x+1,c)}P(Y=0|x+1,c)}$$

$$\times \frac{\frac{P(R=1|Y=0,x+1,c)}{P(R=1|x,c)}P(Y=0|x,c)}{\frac{P(R=1|Y=1,x+1,c)}{P(R=1|x,c)}P(Y=1|x,c)}$$

$$= \frac{P(R=1|Y=1,x+1,c)P(Y=1|x+1,c)}{P(R=1|Y=0,x+1,c)P(Y=0|x+1,c)}$$

$$\times \frac{P(R=1|Y=0,x,c)P(Y=0|x,c)}{P(R=1|Y=1,x,c)P(Y=1|x,c)} = \lambda \exp(\beta_X)$$

where

$$\lambda = \frac{P(R=1|Y=1,x+1,c)P(R=1|Y=0,x,c)}{P(R=1|Y=0,x+1,c)P(R=1|Y=1,x,c)}$$

CRA will therefore give asymptotically unbiased estimates of the OR for X whenever $\lambda = 1$. This result can be seen as a (small) extension of that given by Kleinbaum, Morgenstern and Kupper (Selection bias in epidemiologic studies. *Am J Epidemiol*. 1981;113(4):452-463) for selection bias, with our result additionally allowing for confounders C.

Outcome-dependent missingness

Suppose that missingness is related to outcome Y, but given Y, is independent of X and C, i.e. P(R = 1|X,Y,C) = P(R = 1|Y). Then

$$\lambda = \frac{P(R=1|Y=1)P(R=1|Y=0)}{P(R=1|Y=0)P(R=1|Y=1)} = 1$$

such that CRA is asymptotically unbiased for β_X (and in fact is also asymptotically unbiased for β_C).

Covariate-dependent missingness

Now suppose that missingness is dependent on the covariates, i.e. X and/or C, and given these, is independent of Y, i.e. P(R = 1|X,Y,C) = P(R = 1|X,C). Then

$$P(Y=1|X,C,R=1)=\frac{f(Y=1,X,C,R=1)}{f(X,C,R=1)}$$

$$=\frac{P(R=1|Y=1,X,C)P(Y=1|X,C)f(X,C)}{P(R=1|X,C)f(X,C)}$$

$$=P(Y=1|X,C)$$

such that CRA will give asymptotically unbiased estimates of β_X and β_C, and also β_0 if the study is a cohort design.

Missingness dependent on outcome and confounder

Suppose now that missingness depends on Y and C, but given these is independent of X, i.e., P(R = 1|X,Y,C) = P(R = 1|Y,C). Then,

$$\lambda=\frac{P(R=1|Y=1,c)P(R=1|Y=0,c)}{P(R=1|Y=0,c)P(R=1|Y=1,c)}=1$$

and so CRA will again be asymptotically unbiased for β_X (but in general, will be biased for the other parameters).

Missingness dependent on X and Y

In general, if missingness depends jointly on X and Y (and possibly also C), CRA is biased for β_X.

However, there is a class of mechanisms for which CRA is still asymptotically unbiased for β_X.

Specifically, if P(R = 1|X,Y,C) = s(X,C)t(Y,C) for some functions s(X,C) and t(Y,C), then

$$\lambda = \frac{s(x+1,c)t(Y=1,c)s(x,c)t(Y=0,c)}{s(x+1,c)t(Y=0,c)s(x,c)t(Y=1,c)} = 1$$

and β_X is estimated without bias (asymptotically).

WEB APPENDIX 2

In this appendix we describe the results presented in Table 2 of the main paper regarding what conclusions might be reasonably drawn regarding the missingness mechanism based on fitting a logistic regression model for missingness. We argue under the implicit assumption that both the exposure X and confounders C have independent associations with Y. We first consider cases where only the exposure, the outcome, or some of the confounders are partially observed, and then consider the more complicated case where a combination are partially observed.

Missingness in a confounder

We first suppose that there are missing values in a confounder C_1 and let C_2 denote the other (fully observed) confounders, so that C = (C_1,C_2). Missingness can be investigated by fitting a logistic regression model for the binary observation indicator R (indicating whether C_1 is observed), with X, Y and the other confounders C_2 as covariates. The first part of Table 2 describes the possible results of this analysis. For each possibility, we describe the missingness mechanism(s) which are plausible given the result, and whether the CRA logistic regression exposure OR is asymptotically unbiased.

If the logistic regression model for missingness indicates missingness is associated with X and or C_2, but given these, not Y, missingness can plausibly be assumed to be covariate dependent, and consequently CRA is asymptotically unbiased for the exposure and confounder associations. In a cohort study such an assumption may be quite plausible *a priori* since missingness at study entry can only be associated independently with the future outcome due

to the presence of some other cause of outcome U, not included in the outcome model of interest, which itself affects missingness in the confounder C_1.

Alternatively, if this analysis indicates missingness is independent of exposure X, but depends on Y and possibly components of C_2, the CRA estimate of the exposure OR is again expected to be asymptotically unbiased (missingness dependent on outcome and confounder).

Lastly suppose that we find missingness in C_1 is associated with both X and Y, and possibly the other confounders C_2. In this case, the obvious interpretation is that X and Y both affect missingness in C_1, and unless the mechanism satisfies the special independence condition described earlier, the CRA exposure OR estimate is biased. However, there exist other alternative explanations under which the CRA exposure OR estimate is asymptotically unbiased. Suppose that missingness in C_1 depends on the value of C_1 and X (but not Y). Since C_1 cannot be included in the logistic model for R, an association between R and Y would be a consequence of the effect of C_1 on R and the independent association of C_1 with Y. In this case we would have covariate dependent MNAR missingness, but the CRA would be asymptotically unbiased. A further possibility consistent with the observed data is that missingness depends on Y and C_1 (but not X). Again since C_1 cannot be included in the model for R, the observed association between R and X might be the result of correlation between X and C_1 and the dependence of R on C_1. In this case the CRA exposure OR is again asymptotically unbiased. In this situation contextual knowledge is critical in order to judge which of these scenarios is thought to be plausible, and hence whether the CRA exposure OR is asymptotically unbiased.

Missingness in exposure X

As for the case of missingness in a confounder, if missingness in X appears to be conditionally independent (given the confounders C) of Y, CRA logistic regression is expected to be asymptotically unbiased for both exposure and confounder associations (covariate dependent missingness). If missingness in X is found to be related to Y, but not to the confounders, then an assumption of outcome dependent missingness is plausible, such that the CRA exposure OR estimate is asymptotically unbiased. This conclusion is justified by the fact that if missingness also in truth depended on X, this would (in general) induce an association between R and some of the confounders (conditional on Y), since the confounders are associated with X.

If missingness in X is found to be related jointly to Y and one or more confounders, from the data alone we cannot verify whether the CRA is asymptotically unbiased for the exposure OR. The conclusion we would most obviously draw is that missingness depends on outcome Y and confounders C, such that the exposure OR is asymptotically unbiased. However, alternative explanations are also quite possible, under which the association is biased. One is that missingness depends on X and Y, and the observed association between R and some of the Cs is due to the effect of X on R and the correlation between X and these Cs. In this case, the CRA exposure OR is generally biased. Alternatively, missingness could depend on X and C, and the observed association between R and Y (conditional on C) is induced by the independent association between X and Y and the effect of X on R. In this case of covariate dependent missingness, the CRA is asymptotically unbiased. Thus again, in this situation, contextual knowledge is critical to determining which missingness assumption is plausible.

Outcome missingness

In this setting we can investigate whether missingness in the outcome is related to X and/or C. Suppose first that we find that missingness in Y is associated with X, but not C. In this case, it would usually be reasonable to assume that missingness depends only on X, such that the CRA is asymptotically unbiased for the exposure and confounders associations. Such a conclusion is justified by the fact that if missingness also depended on Y, this would induce an association between R and one or more of the confounders.

If missingness is found to be independent of X conditional on C, this suggests an assumption of missingness dependent on outcome and confounder is reasonable, such that the CRA exposure OR is again asymptotically unbiased.

If missingness in Y is found to be associated with X and C the natural interpretation is that missingness is independent of Y, conditional on X and C (covariate dependent missingness), such that the CRA is asymptotically unbiased. However again there are alternative explanations consistent with the observed data. Missingness could depend on Y and C, and the independent association between X and Y induces an association between R and X (conditional on C). In this case the CRA exposure OR is asymptotically unbiased. Alternatively, missingness could depend on X and Y, with the independent association of C with Y inducing the association between R and C (conditional on X). In this case the exposure OR is generally estimated with bias. Thus again here contextual knowledge is essential in order to gauge the plausibility of the different assumptions.

Missingness in multiple variables

The preceding considerations focused on situations where only one variable (or a block of variables which are either all observed or all missing) contains missing values. In reality studies often suffer from missing values in multiple variables involved in the outcome model. This inevitably complicates the process of investigating the missingness mechanisms and thereby judging whether the CRA is likely to give asymptotically unbiased exposure OR estimates. A simplification that may be possible is that if the rate of missingness in a variable is very low (e.g. less than 5%), one might choose to essentially assume that it is fully observed for the purposes of investigating the missingness mechanisms of the other partially observed variables and for deciding whether the CRA logistic regression exposure OR is asymptotically unbiased.

Suppose first that multiple confounders are partially observed, but that the exposure and outcome are fully observed. In this case we can treat the partially observed confounders as a vector C_1 and proceed as described previously for a single partially observed confounder, thereby ignoring in our investigation of missingness any observed values in components of C_1 when at least one component is missing.

Now suppose that two or more of X, Y and C are partially observed. If C is partially observed, we again divide C into those partially observed (C_1) and those which are fully observed (C_2). In some situations it may be reasonable to assume that the different missingness processes are independent, conditional on X, Y and C. In this case, the CRA exposure OR is asymptotically unbiased provided each missingness mechanism falls within one of the classes described previously. The problem thus reduces to determining whether each mechanism falls within one

of the described classes, and each missingness mechanism can be investigated separately as previously described for the case of missingness in one variable.

Unfortunately a difficulty which then arises is that an analysis of missingness in one variable suffers from missingness in the other variable(s). There are a myriad of possibilities for what analyses of missingness might indicate in this situation and what conclusions we might reasonably draw (if any) from them. As an example, suppose we have missingness in X and confounders C_1. In this case a logistic regression model for the observation indicator R_X with covariates Y and C will exclude those records with at least one confounder missing, leading to a loss of precision and potentially bias (in the estimates of the model for R_X), unless R_X and R_{C1} are conditionally independent given Y and C. Nevertheless, if this CRA of missingness suggested missingness in X was conditionally independent of Y conditional on C, it may be reasonable to assume R_X depends only on C. Such a conclusion may be reasonable since if the exclusion of those records with confounders missing from the analysis for R_X induces bias, it is arguably unlikely to cause bias such that R_X and Y appear conditionally independent given C if in truth they are not.

The preceding considerations demonstrate that with missingness in multiple variables the investigation of missingness using the observed data becomes considerably more difficult. In consequence, study specific contextual knowledge regarding missingness mechanisms becomes commensurately more important. Nevertheless, we believe a combination of careful analysis of the observed data and contextual considerations can often give sufficient credibility for a particular assumption about missingness.
